# Small Changes, Big Gains: A Quality Improvement Approach to Increasing Responsive Care for Infants and Toddlers with Cancer on the Inpatient Unit

**DOI:** 10.3390/children13020207

**Published:** 2026-01-31

**Authors:** Jennifer L. Harman, Alyssa Marchetta, David Wittman, Niki Jurbergs

**Affiliations:** 1 Department of Family and Preventive Medicine, University of Arkansas for Medical Sciences, Little Rock, AR 72205, USA; 2 Department of Psychology and Biobehavioral Sciences, St. Jude Children’s Research Hospital, Memphis, TN 38105, USA; 3 Office of Quality and Patient Safety, St. Jude Children’s Research Hospital, Memphis, TN 38105, USA

**Keywords:** infants, toddlers, inpatient, responsive care, pediatric cancer, quality improvement

## Abstract

**Highlights:**

**What are the main findings?**

- The provision of written psychoeducation to caregivers resulted in a significant decrease in nursing shifts during which young children were not spoken to or held.

**What is the implication of the main finding?**

- Quality improvement methods and low-cost interventions can help promote responsive caregiving for young patients during inpatient admissions, supporting development and overall wellness.

**Abstract:**

Background: Responsive caregiving supports infant and toddler wellbeing. Yet, based on nursing observational data, a significant number of one institution’s inpatient infant and toddler patients with cancer—who are uniquely vulnerable due to the developmental risks associated with their illness and treatment—were not spoken to or held by their caregiver at any time when nursing was present over the course of day shifts. Objective: This clinical quality improvement project aimed to increase caregiver engagement in responsive interactions during inpatient stays. Methods: The Model for Improvement framework was used. Implementation, evaluation, and reporting followed the SQUIRE 2.0 framework. Root causes were analyzed with fishbone and key driver diagrams. Outcomes were tracked with control charts and percentage of nursing shifts during which responsive care was not observed. Statistical process control was used to study interventions. Results: Two intervention cycles were completed and resulted in significant and meaningful (>1 sigma) reductions in nursing shifts during which infants and toddlers were not spoken to or held. Conclusions: Caregiver psychoeducation interventions increased responsive care of infants and toddlers in our oncology inpatient setting. This low-cost intervention may be adaptable across inpatient settings.

## 1. Introduction

Responsive caregiving, or when a caregiver notices, interprets, and appropriately responds to a young child’s cues, is essential for infant and toddler development. These responsive interactions foster secure attachment styles, strengthen early brain development, and support emotional regulation and coregulation during the critical first three years of life [1,2]. In fact, young children who experience consistent, sensitive caregiving demonstrate significantly better cognitive, social, and emotional outcomes than those who experience less responsive caregiving [3]. For young children undergoing medical treatment associated with cognitive or other developmental risks (e.g., central nervous system directed treatment, prolonged hospitalizations, repeated exposure to anesthesia, etc.), the provision of responsive caregiving may be a particularly important tool in the mitigation of developmental risk and the promotion of resilience.

Despite its importance, responsive caregiving may be disrupted in hospital settings for many reasons. For example, when a young child is hospitalized, parental stress related to the child’s condition and treatment demands is common [4]. Higher parental stress is linked to less responsive, less consistent, and more strained parent-young child interactions outside of the inpatient setting [5,6], and, more specifically, with less parental engagement within the hospital setting [7]. Similarly, parents may feel uncertain about their role. Some believe they must remain quiet, interact less often, or defer caregiving to nursing staff when their child is medically fragile [8,9]. Additionally, the inpatient setting is fast-paced and driven by medical urgency and staff routines, not by the child’s or family’s routines [4]. Among children being treated for cancer, treatment side effects, infection precautions, and the intensity of care may also limit opportunities for natural caregiver–child interaction. Without intentional support, opportunities for connection such as talking, singing, holding, or participating in daily caregiving tasks may be overlooked. These missed opportunities can contribute to disruptions in early relational health, a significant concern given the long-term developmental vulnerabilities of children with serious illnesses [10].

At our institution, observational baseline data were gathered through direct communication with nursing staff assigned to infant and toddler oncology patients on the inpatient units during day shifts. Authors of this paper collaborated with nurses to document caregiver behaviors, specifically noting whether caregivers were observed speaking to or holding their child at any point during the shift. More specifically, at the end of each day shift (*N* = 5) over a 5-day period, nurses dichotomously indicated whether they observed specific responsive care behaviors at any point when they were is a young patient’s room. These data revealed that caregivers were observed speaking to their child during only 35% of nursing shifts and holding their child during approximately half of the shifts, highlighting a significant gap in responsive caregiving practices [11]. These data underscored a critical practice gap. While psychosocial standards of care in pediatric oncology emphasize family-centered and developmentally supportive care [10], there was no systematic process in our setting for ensuring that caregivers received guidance and support for engaging in responsive caregiving during hospitalization [11].

Quality improvement (QI) methods provide a framework for addressing such system-level practice gaps. The Model for Improvement emphasizes iterative testing of small changes to improve outcomes in complex health care settings [12]. QI approaches have been successfully applied to enhance patient safety, care efficiency, and family-centered practices across pediatric subspecialties. In the domain of developmental and psychosocial care, low-cost, scalable interventions that embed evidence-based practices into routine care can yield meaningful improvements for patients and families [13]. Embedding psychoeducation on responsive caregiving into standard inpatient practice represented a promising strategy to align daily caregiver–child interactions with developmental science.

Our multidisciplinary team designed and implemented a QI initiative to increase caregiver engagement in responsive care for infants and toddlers with cancer who were being cared for in the inpatient setting. Guided by the Model for Improvement, the project focused on systematically delivering psychoeducation about responsive caregiving through multiple modalities (e.g., electronic health record (EHR) portal messages, inpatient television screensavers, caregiver handouts). The specific aim was to decrease the percentage of nursing shifts during which hospitalized infants and toddlers were not observed to be spoken to or held by their caregiver, while balancing feasibility and staff burden.

This manuscript describes the development, implementation, and outcomes of this QI initiative. We hypothesized that systematic delivery of psychoeducation to caregivers would increase their engagement in responsive caregiving behaviors during hospitalization. By increasing caregiver understanding of the benefits of talking, singing, holding, and other responsive interactions, the project sought to promote infants’ and toddlers’ developmental wellbeing during cancer treatment. Because the intervention is low-cost, sustainable, and grounded in family-centered care principles, it has the potential to be adapted across pediatric settings [2,3].

## 2. Methods

### 2.1. Context

This initiative took place at St. Jude Children’s Research Hospital (SJCRH), the only National Cancer Institute-designated Comprehensive Cancer Center devoted solely to children. SJCRH has 77 inpatient beds and routinely admits infants and toddlers (<36 months of age) for medical care during their cancer treatment.

A multidisciplinary “Responsive Care Team” was created with the ultimate goal of increasing and improving responsive care for our youngest patients. The team began by attempting to characterize current state; specifically, exploring the frequency of developmental responsive care parents and other primary caregivers delivered to infants and toddlers during inpatient admissions, and to address any identified practice gaps. The Responsive Care Team included caregiver representation and members from the following hospital services and departments: Inpatient Nursing, Nursing Education, Pediatric Intensive Care Unit, Respiratory Therapy, Rehabilitation Services, Psychosocial Services, Office of Quality and Patient Safety, and Patient and Family Experience Office.

This clinical quality improvement initiative used the Model for Improvement [12] to guide development of the problem statement, small tests of change, and iterative learning cycles. Implementation, evaluation, and reporting were structured using the SQUIRE 2.0 standards, which provide a framework for clearly and systematically reporting quality improvement work in healthcare, emphasizing context, intervention details, study design, and outcomes to enhance transparency and reproducibility [13].

### 2.2. Inclusion/Exclusion

All patients younger than 36 months of age during the project period who were admitted to the oncology, transplant or pediatric intensive care units were included. In several cases, it was inappropriate to share the written education materials that were created with caregivers with limited English proficiency (LEP). In these instances, the standard workflow was modified to make the intervention available to all families (e.g., phone call with help of interpreter; in-person visit with help of interpreter, etc.). As such, LEP did not result in exclusion.

### 2.3. Intervention

**Figure 1:**
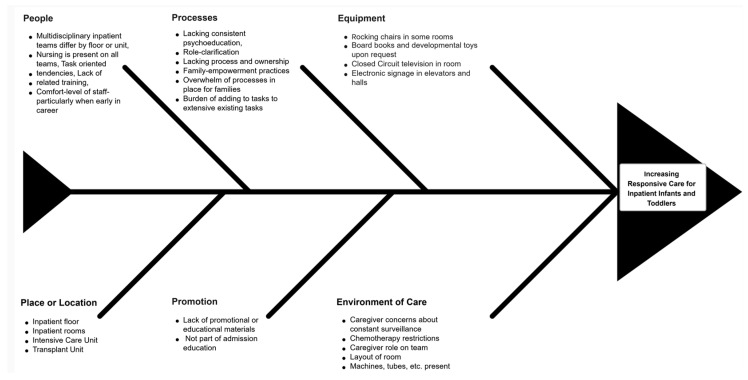
Fishbone (Ishikawa) diagram of hypothesized contributors to lack of responsive caregiving on the inpatient oncology unit (caregiver, staff, workflow/technology, environment, and clinical status domains).

**Figure 2:**
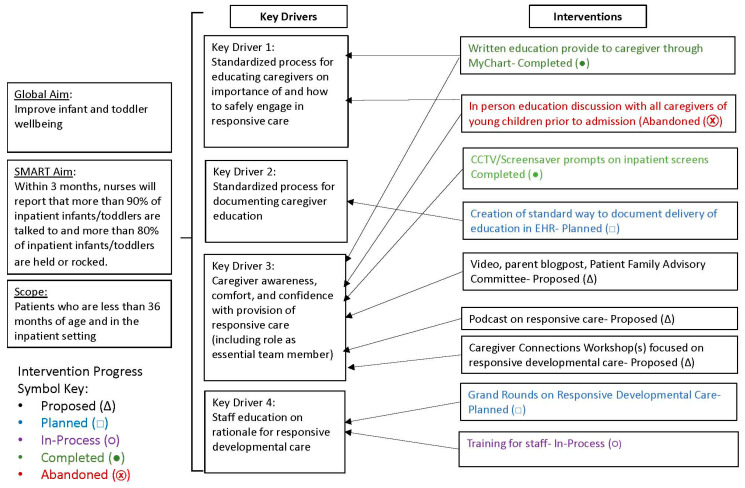
Key driver diagram linking primary drivers to change ideas to decrease observations where caregivers were not talking/singing, holding/comforting, and connecting during routine care.

**Rationale and theory of change.** Potential root causes for the high frequency of nursing shifts in which responsive care behaviors were not observed were examined using categorizations on a fishbone diagram (Figure 1) and a key driver diagram (Figure 2). The fishbone diagram helped the team systematically identify and organize contributing factors across domains such as environment, processes, people, and equipment. The key driver diagram was used to visually map the relationships between the project aim, primary drivers, and specific change ideas, guiding the selection and prioritization of interventions most likely to influence caregiver engagement in responsive behaviors [14]. Each of these diagrams suggested that brief, standardized psychoeducation and visual prompts could increase caregiver engagement in interactive responsive caregiving behaviors (e.g., talking, singing, holding, connecting during routine care) during hospitalization. Therefore, interventions focused on the provision of brief psychoeducation coupled with visual prompts.

**Figure A1:**
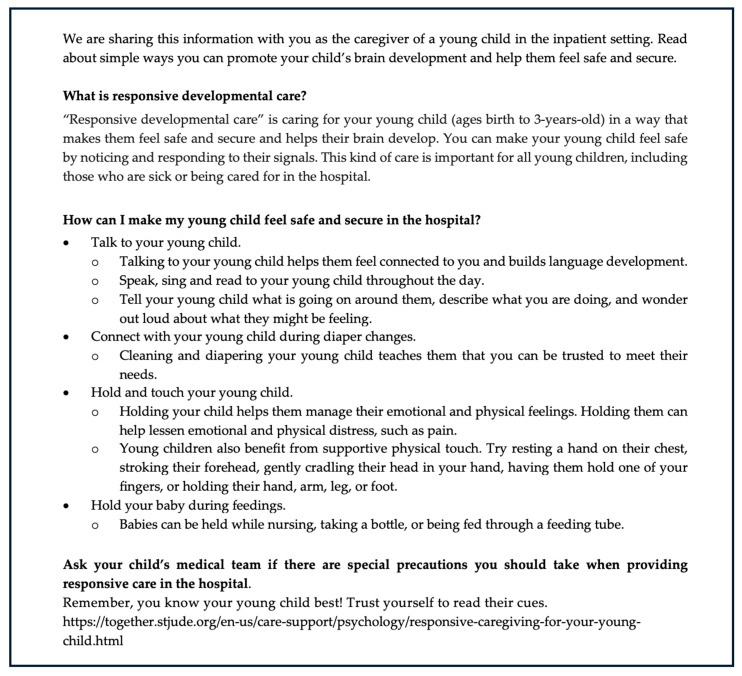
EHR Portal Message with Responsive Care Education.

**Core components and delivery.** The first intervention was the delivery of standard education via a message sent to caregivers in the patient’s electronic health record portal (i.e., EPIC MyChart message) at the time of admission (Appendix A, Figure A1). The message introduced responsive developmental care in simple, easy to understand language, briefly noted why responsive caregiving is important for brain development, and encouraged caregiver engagement in specific behaviors (e.g., speaking to their child, holding their child, etc.). A link to a webpage with additional information about responsive caregiving was also included. The information in the portal message was also formatted into a one-page handout. In the event that a clinician from Psychology met with a family during admission, a hard copy of this handout was shared with them.

**Figure A2:**
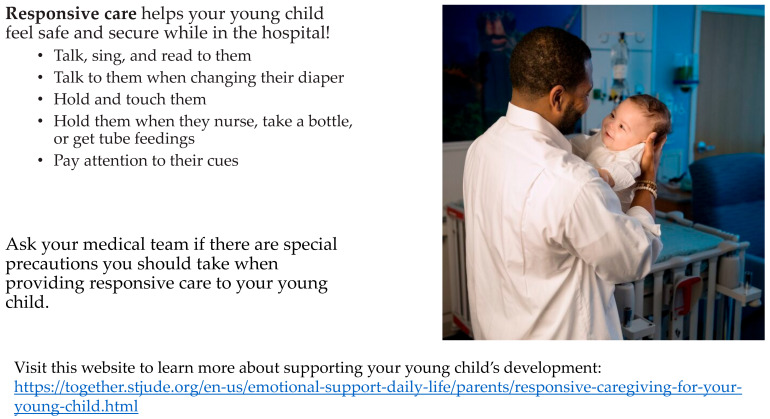
Hospital Room TV Image with Responsive Care Education.

The second intervention was the display of an educational television screensaver in all inpatient rooms. The screensaver (Appendix A, Figure A2) included a briefer version of the content in the portal message and was designed to serve as a visual prompt or reminder of the portal message. Additionally, the screensaver image included a QR code that took caregivers to the same webpage linked in the portal message.

### 2.4. Plan-Do-Study-Act Cycles and Adaptations

Two sequential Plan-Do-Study-Act (PDSA) cycles were completed. A PDSA cycle is a repetitive four-step process for testing improvements in healthcare settings, where teams plan a specific change, carry it out on a limited basis, evaluate the outcomes, and adjust the strategy accordingly. This method supports evidence-based refinement and ongoing enhancement of systems [14]. The first cycle focused on delivery of the portal message and handout distribution. The second cycle launched the screensaver visual prompt. Between cycles, the team analyzed control charts to monitor trends and distinguish common-cause variation from special-cause signals [14].

### 2.5. Study of the Intervention

An observational quality improvement design with continuous monitoring and statistical process control (SPC) was used to study interventions. The team pre-specified special-cause rules (e.g., centerline shifts, trends) to determine when system performance changed. SPC charts and qualitative implementation notes were reviewed in regular meetings to support rapid learning and small adaptations.

### 2.6. Measures

Primary outcomes were nurse-observed occurrences of caregiver delivered responsive care behaviors. Inpatient nurses received in-person training followed by brief written guidance on identifying instances of caregivers (1) talking or singing to their child and (2) holding or rocking their child. At the end of each day shift (i.e., 7:00 am–7:00 pm), Responsive Care Team members messaged nurses via the hospital’s secure chat platform to ask if either of these behaviors had been observed during the shift. Nurses responded “yes” if the behavior was observed at least once during the shift and “no” if the behavior was not observed during the shift. The number of times a nurse entered each inpatient room and the amount of time a nurse spent in each inpatient room varied. All data were de-identified prior to analysis and aggregated at the shift or day level. Daily observation data collection continued until a centerline shift was noted on a control chart.

Process data for the EHR portal message were extracted from routine operational reports (i.e., message sent and message opened by caregiver). Screensaver uptime was confirmed via spot checks.

### 2.7. Missing Data

Shifts lacking an observation recording from nursing were treated as missing and excluded from denominators for SPC. Consistent with quality improvement monitoring practices, no imputation of data was performed.

### 2.8. Analysis

P-charts were plotted for each primary outcome (i.e., how many shifts nurses did not observe caregivers talking to or holding their children) with subgroups at the shift (or day-aggregated) level. Baseline centerlines (i.e., average rates before any changes were made) and control limits (i.e., normal range of variation) were estimated from the pre-intervention period and recalculated following special-cause signals. Centerline shifts, trend rules, and magnitude of improvement (e.g., >1-sigma reduction in the percentage of shifts during which patients were **not** spoken to or **not** held) were used as evidence of special cause. Annotations marked PDSA cycle start dates and key changes.

### 2.9. Ethical Considerations

The institutional review board at St. Jude Children’s Research Hospital determined that the initiative did not meet the definition of human subjects’ research. Rather, it was a clinical quality improvement practice change. No individual-level consent was required for de-identified, aggregate monitoring

### 2.10. Patient, Caregiver, and Staff Involvement

A caregiver representative from our institution’s Patient and Family Advisory Council served on the Responsive Care Team, contributing to the design of interventions including language in the educational materials. Nursing, psychology, child life, rehabilitation, and quality and safety staff co-developed workflows for all interventions and provided ongoing feedback for iterative refinement.

### 2.11. Implementation and Sustainment

To support sustainment, the portal message was embedded into admission workflows and handout distribution was assigned to defined roles, and the screensaver was set to cycle continuously with periodic refreshes.

## 3. Results

### 3.1. Process Measure

Eighty-one percent (81%) of in-scope caregivers received and viewed the portal message. Screensaver uptime was confirmed in 100% of spot checks.

### 3.2. Primary Outcomes

**Figure 3:**
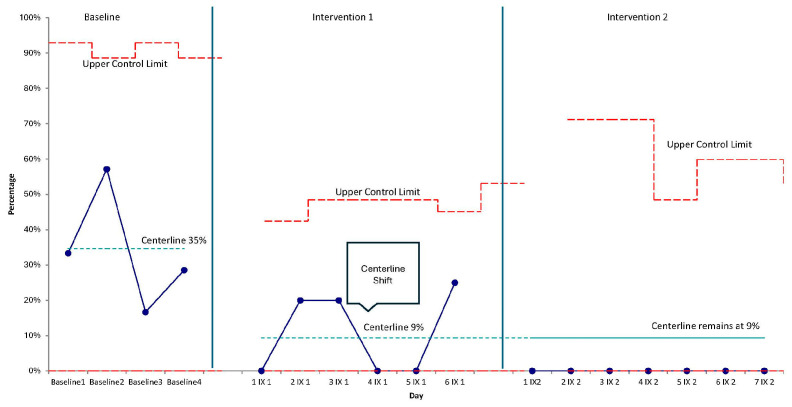
Statistical process control (p-chart) of the percentage of nursing shifts during which caregivers were **not** observed talking/singing to their child, with PDSA cycle annotations and centerline shifts.

Implementation spanned a total of 13 nursing shifts. Following implementation, the percentage of nursing shifts during which caregivers were not observed to speak or sing to their child decreased markedly. The p-chart shows a sustained series of points below the baseline centerline after the first PDSA cycle, prompting a downward centerline shift. Performance remained improved through the second cycle with values clustering near zero across the post-implementation period (Figure 3). These signals indicate special-cause improvement (i.e., >1 sigma) in the desired direction.

**Figure 4:**
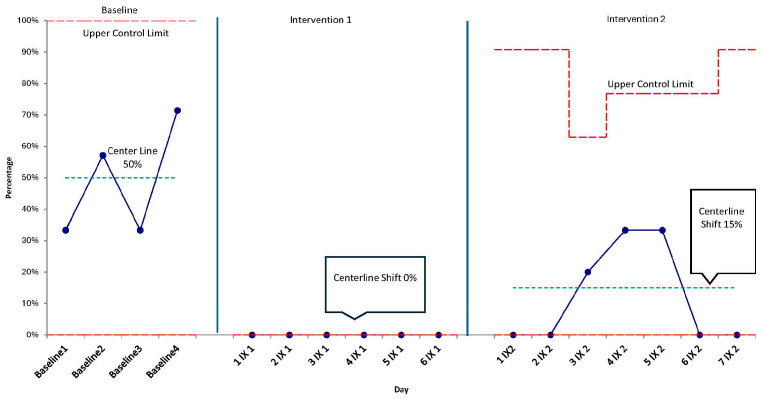
Statistical process control (p-chart) of the percentage of nursing shifts during which caregivers were **not** observed holding/rocking their child, with PDSA cycle annotations and centerline shifts.

Similarly, the percentage of nursing shifts during which caregivers were not observed to hold or rock their child decreased over time. The control chart demonstrates a post-intervention run below the baseline centerline with a subsequent centerline shift downward (Figure 4), consistent with special-cause improvement (i.e., >1 sigma). This represents a centerline shift from 50% during baseline to a centerline of 15% in post period, equating to less than one out of five shifts without holding in the post period.

### 3.3. Overall Pattern Across Outcomes

Across both outcomes, SPC signals (sustained runs and centerline shifts) support that the changes were unlikely due to common-cause variation. The directionality was consistent—fewer shifts with caregivers **not** observed talking/singing to their child and fewer shifts with caregivers **not** observed holding their child. This aligned with the project’s aim to increase responsive caregiving through caregivers talking/singing to their child and holding/rocking their child during inpatient oncology stays.

## 4. Discussion

This quality improvement initiative successfully utilized the systematic provision of caregiver education to achieve our objective: increase the delivery of responsive care, a well-supported intervention to promote child development, to infants and toddlers being treated for cancer in the inpatient setting. Importantly, the educational interventions employed represent low cost, low effort, and potentially high impact actions most medical settings could reasonably offer to families of young patients in the context of a wide range of risk factors (e.g., medical diagnoses, developmental delays, etc.).

While not formally measured, additional, unplanned benefits of the interventions employed were appreciated during the course of the project. For example, bedside nurses reported and exhibited increased awareness of the benefits of responsive care as well as increased comfort and confidence in educating and modeling responsive care for parents in the course of typical nursing care. Related, this increased awareness and comfort in Inpatient Nursing appeared to serve as an accelerant, increasing awareness and even prioritization of responsive care across clinical disciplines. Anecdotally, members of the Responsive Care Team observed organically increased discussion of patient-caregiver interactions during inpatient medical rounds, coupled with treatment planning targeting increased responsive caregiving (e.g., nursing-led education for caregivers, referrals to Psychology and Child Life to provide caregiver education and support). Ultimately, our team observed what might be described as a culture shift across the hospital in which clinicians from multiple disciplines are modeling ownership of the responsibility to educate and support families in responsive caregiving. Such a shift is significant because responsive caregiving is foundational for children’s brain development, attachment, and cognitive, social, and emotional outcomes [1,2,3]. Partnering with parents and valuing family involvement, such as teaching and encouraging responsive caregiving, has been shown to enhance the quality of care delivered [15,16]. Furthermore, high work engagement among medical providers, which can be promoted by engaging in or modeling responsive caregiving, is associated with reduced stress levels among pediatric nurses [17].

The Responsive Care Team continues implementing interventions aimed at increasing responsive care for our young patients. Currently, we continue providing psychoeducation through EHR messages, printed handouts, and TV prompts. We plan to adapt these resources for caregivers with limited English proficiency and for those with lower literacy, maximizing accessibility for all families. We also aim to strengthen nurse training on how to prompt, model, and encourage responsive caregiving, not only in inpatient units but also in outpatient clinics and ancillary areas. In addition, we hope to collaborate with nursing to establish systematic support for caregivers who are hesitant to hold or touch their child due to medical status or perceived fragility. Further, to reinforce these strategies, we will ensure that responsive caregiving principles are discussed by psychologists and other psychosocial professionals during every new patient consult for children <36 months of age. Additionally, our institution plans to implement a weekly developmental group led by psychosocial professionals (e.g., psychology, child life, music therapy) to model responsive caregiving through play and music. Long-term, we aim to demonstrate improvements in the frequency and quality of responsive caregiving and to evaluate its impact on children’s developmental outcomes.

This project has several important limitations to keep in mind when interpreting the findings and considering possible future applications in other settings. The outcome measure relied on nurse-observed, binary (yes/no) identification of responsive caregiving behaviors once per 12 h shift, which introduces several sources of potential variability in how different nurses noticed and interpreted caregiver behavior. The absence of an observed behavior does not necessarily mean it did not occur at all throughout the nursing shift. These factors limit the precision of the data and may underrepresent actual caregiving patterns. There is also the possibility of observer effects, where caregivers or nurses may have changed their behavior or their noticing of behavior simply because they were aware of the initiative. Although this may have influenced the results, such shifts are still meaningful within a quality improvement context, as increased attention to the targeted behavior is itself an intended outcome. Finally, several contextual strengths at our institution, such as strong nursing engagement, a family-centered culture, reliable EHR messaging systems, and supportive family presence policies, may not be present in all inpatient settings, which should be considered when thinking about generalizability.

## 5. Conclusions

Our quality improvement initiative demonstrates that even small interventions—such as delivering psychoeducation through electronic medical record messages, printed handouts, and TV prompts—can significantly enhance responsive caregiving on the inpatient unit. Given that the highest incidence of childhood cancer occurs between birth and age four [18], and that children in this age group are particularly vulnerable to developmental and cognitive challenges due to intensive treatment, side effects, stress responses, and limited access to enriched environments [19,20], the need for protective intervention is urgent. Responsive caregiving has been shown to buffer against these risks, and our approach offers a cost-effective, low-effort strategy to support families during this critical window of brain development. These children and their families deserve nothing less.

## Data Availability

Given these data are clinical data (as opposed to research data) and that they were solely used for quality improvement purposes related to a clinical practice change, they are not available for sharing.
